# Risk factors for primary graft dysfunction after heart transplantation—a systematic review and meta-analysis

**DOI:** 10.1016/j.jhlto.2026.100483

**Published:** 2026-01-30

**Authors:** Mats T. Vervoorn, Selma E. Kaffka genaamd Dengler, Elisa M. Ballan, Jord F. Kernkamp, Mudit Mishra, Sue A. Braithwaite, Marish I.F.J. Oerlemans, Niels P. van der Kaaij

**Affiliations:** a University Medical Center Utrecht, Department of Cardiothoracic Surgery, Division of Heart & Lungs, Utrecht, Netherlands; b University Medical Center Utrecht, Department of Cardiology, Laboratory of Experimental Cardiology, Division Heart & Lungs, Utrecht, Netherlands; c Netherlands Heart Institute, Utrecht, Netherlands; d University Medical Center Utrecht, Department of Anesthesiology, Division of Vital Functions, Utrecht, Netherlands; e University Medical Center Utrecht, Department of Cardiology, Division Heart & Lungs, Utrecht, Netherlands

**Keywords:** Heart failure, Primary graft dysfunction, Heart transplantation

## Abstract

**Background:**

Primary graft dysfunction (PGD) is an important contributor to early mortality after heart transplantation (HTX). The International Society for Heart and Lung Transplantation (ISHLT) published a consensus definition of primary graft dysfunction in 2014. We conducted a systematic review and meta-analysis of published literature to identify risk factors for primary graft dysfunction according to this consensus definition.

**Methods:**

According to PRISMA-guidelines, eligible studies were identified using Medline, Embase, and Cochrane. Studies were included if focused on patients ≥18 years old who received a primary, isolated HTX and outcome was specified as PGD according to the ISHLT consensus definition. Risk factors for severe PGD were included in meta-analysis if reported in ≥2 studies.

**Results:**

A total of 39 studies were included. Significant heterogeneity was noted. A total of 37 risk factors for PGD were identified. Meta-analysis identified recipient amiodarone therapy, female sex of the donor, recipient prior sternotomy (other than for LVAD implantation), recipient LVAD therapy and cold ischemic time per hour increment as risk factors for severe PGD. Blood product administration was associated with severe PGD in multiple studies, but was excluded from meta-analysis due to heterogeneity in definition.

**Conclusion:**

In this systematic review, we identified a total of 37 risk factors for PGD, while meta-analysis solidified amiodarone therapy, female sex of the donor, recipient prior sternotomy (other than for LVAD implantation), recipient LVAD therapy and cold ischemic time per hour increment as risk factors for severe PGD. Blood product administration was associated with severe PGD in multiple studies, but was excluded from meta-analysis due to heterogeneity in definition.

Heart transplantation (HTX) is the current golden standard treatment for end-stage heart failure, with over 6000 transplants conducted annually worldwide.^1^ Although overall survival has improved over time, 1-year mortality remains relatively high, with primary graft dysfunction (PGD) being a significant contributor.^1^, ^2^, ^3^, ^4^, ^5^

Prior to the International Society for Heart and Lung Transplantation's (ISHLT) publication of a consensus definition for PGD in 2014,^6^ different definitions were used by different HTX centers, limiting the ability to compare outcomes and conduct research. According to this consensus definition, PGD must be diagnosed within 24 hours after HTX and distinguished from secondary graft dysfunction, such as immune-mediated rejection, pulmonary hypertension, or surgical complications. PGD can be further categorized as left (PGD-LV) or right ventricular (PGD-RV), with varying levels of severity based on echocardiographic and hemodynamic parameters, including dependency on inotropes or mechanical circulatory support for survival (Table 1).^6^

**Table 1:** Classification of Primary Graft Dysfunction According to the International Society for Heart and Lung Transplantation

1. PGD-Left ventricle (PGD-LV):	Mild PGD–LV: One of the following criteria must be met:	I. LVEF ≤ 40% by echocardiography, or Hemodynamics with RAP > 15 mm Hg, PCWP > 20 mm Hg, CI < 2.0 L/min/m^2^ (lasting more than 1 h) requiring low-dose inotropes
	Moderate PGD-LV: Must meet one criterion from I and another criterion from II:	I. One criterion from the following: a. LVEF ≤ 40% by echocardiography, or b. Hemodynamic compromise with RAP > 15 mm Hg, PCWP > 20 mm Hg, CI < 2.0 L/min/m^2^, hypotension with MAP < 70 mm Hg (lasting more than 1 h) I. One criterion from the following: a. High-dose inotropes—Inotrope score > 10^a^ or b. Newly placed IABP (regardless of inotropes)
	Severe PGD–LV	I. Dependence on left or biventricular mechanical support including ECMO, LVAD, BiVAD, or percutaneous LVAD. Excludes requirement for IABP.
2. PGD-right ventricle (PGD-RV):	Diagnosis requires either both I and II, or III alone:	I. Hemodynamics with RAP > 15 mm Hg, PCWP < 15 mm Hg, CI < 2.0 L/min/m^2^ II. TPG <15 mm Hg and/or pulmonary artery systolic pressure < 50 mm Hg III. Need for RVAD

BiVAD, biventricular assist device; CI, cardiac index; ECMO, extracorporeal membrane oxygenation; IABP, intra-aortic balloon pump; LV, left ventricle; LVAD, left ventricular assist device; LVEF, left ventricular ejection fraction; PCWP, pulmonary capillary wedge pressure; PGD, primary graft dysfunction; RAP, right atrial pressure; RVAD, right ventricular assist device; TPG, transpulmonary gradient

a Inotrope score = dopamine (×1) + dobutamine (×1) + amrinone (×1) + milrinone (×15) + epinephrine (×100) + norepinephrine (×100)67 with each drug dosed in μg/kg/min.

Although several observational cohort studies have identified risk factors for PGD, no systematic review or meta-analysis solely aimed at identifying risk factors for PGD according to the consensus definition has been performed. The objective of this study is to identify risk factors for PGD according to the 2014 ISHLT consensus definition by systematic review of the literature that has been published since.

## Methods

### Search strategy

This systematic review adhered to the Preferred Reporting Items for Systematic Reviews and Meta-Analyses (PRISMA) guidelines. Eligible studies were identified through a comprehensive search of Medline, Embase, and Cochrane databases until September 1, 2024. The search strategy involved using a combination of free-text terms in Title/Abstract and subject indexing of keywords, such as "Heart Transplantation" and "Primary Graft Dysfunction," and their synonyms. We excluded papers published before the ISHLT consensus conference on April 23, 2013, and filtered results to exclude conference proceedings, comments, and case reports. In addition, reference lists of included studies were analyzed for additional studies. The full search string is displayed in the supplementary files (Appendix S1).

### Study selection

**Figure 1:**
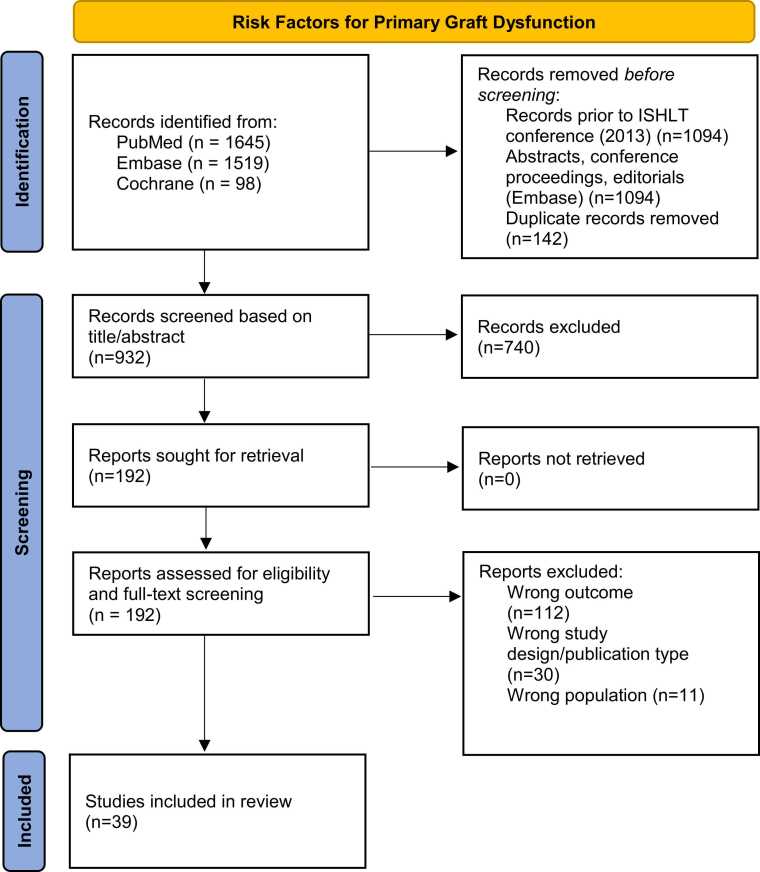
PRISMA flow-diagram of the conducted search

Studies were included if the population consisted of adults (≥18 years) who received a primary, isolated HTX, and outcome was specified as the development of PGD according to the ISHLT consensus definition. Reported risk factors in each study had to be identified by multivariate analysis for inclusion. Exclusion criteria included studies that involved results from univariate analysis only, pediatric HTX (<18 years), retransplantation, multi-organ transplants, and non-English literature. The conducted search is highlighted in Figure 1.

Identified articles were uploaded into the Mendeley Reference Manager (Mendeley, London, United Kingdom) for duplicate removal and uploaded to Rayyan.ai for further processing. Two researchers (M.V., J.K.) independently conducted title/abstract screening and full-text analysis, with disagreements resolved through discussion and, if necessary, consultation with a third researcher not involved in the search and selection process (SKgD).

### Quality assessment

We used the Quality in Prognosis Studies tool (QUIPS) for quality and bias assessment of the included observational studies,^7^ and the Risk of Bias 2 (RoB2) for the single randomized, controlled trial.^8^ The tools include multiple items that are judged separately and based on the ratings of the included items, a conclusive judgment regarding risk of bias within each domain is made and expressed on a 3-grade scale (high, moderate, or low risk of bias). The process of quality assessment was conducted independently by 2 separate researchers (M.V., E.B.). Disagreements were resolved by discussion and, if necessary, consultation of a third researcher (S.K.g.D.).

### Data extraction

For each study included in the analysis, the following data were extracted and recorded into a pre-specified data extraction file in Microsoft Office Excel 2016: author, year of publication, study design, geographic location, recruitment period, population, exclusion criteria, total participants, mean age, percentage of female patients, durable left ventricular assist device (LVAD) prior to HTX, recipient extracorporeal membrane oxygenation (ECMO) prior to HTX, overall incidence of any form of PGD, incidence of mild, moderate, severe, moderate-severe and RV-PGD, recipient, donor and procedural risk factors with corresponding odds ratio’s and confidence intervals.

### Statistical analysis

For meta-analysis, only risk factors that were reported in ≥2 studies assessing severe PGD were included. The decision to pursue meta-analysis for severe PGD was based on clinical relevance. We did not pool heterogeneous data or transform original data to improve homogeneity for the purpose of meta-analysis. We used Review Manager Software, version 5.4 (Cochrane Collaboration, Oxford, UK) to calculate pooled odds ratios (OR) and 95% confidence intervals (CI) for each risk factor using the inverse variance method with a DerSimonian and Laird random-effects model. A random-effects model was chosen due to the expected heterogeneity in the identified studies. The ORs were based on the natural logarithm of reported ORs and calculated standard errors. In case of multiple reports from overlapping datasets, only the study with the largest representative sample size was included in the meta-analysis.

## Results

### Study characteristics

After duplicate removal, a total of 932 original studies were retrieved for title/abstract screening. After exclusion of 740 studies, a total of 192 studies were subjected to full-text analysis. During full-text analysis, 112 were excluded due to wrong outcome, 30 due to wrong study design or publication type (including univariate modeling only), and 11 due to wrong study population. No additional studies were identified after analysis of the reference lists of all included studies, resulting in a total of 39 articles to be included in our systematic review (Figure 1).^9^, ^10^, ^11^, ^12^, ^13^, ^14^, ^15^, ^16^, ^17^, ^18^, ^19^, ^20^, ^21^, ^22^, ^23^, ^24^, ^25^, ^26^, ^27^, ^28^, ^29^, ^30^, ^31^, ^32^, ^33^, ^34^, ^35^, ^36^, ^37^, ^38^, ^39^, ^40^, ^41^, ^42^, ^43^, ^44^, ^45^, ^46^ Characteristics of included studies are highlighted in Table 2.

**Table 2:** Characteristics of Included Studies

Author	Year	Study design	Location	Recruitment period	Total participants (n)	Age (years)	Female sex (%)	Recipient LVAD (%)	Recipient ECMO (%)	Total PGD (%)	Mild PGD (%)	Moderate PGD (%)	Severe PGD (%)	Moderate/severe PGD (%)	RV PGD (%)
Avtaar Singh	2019	Prospective MC	UK	2012-2015	450	45.3	32.7	33	1.3	163/450 (36.2)	NR	NR	NR	NR	NR
Avtaar Singh	2019	Retrospective MC	UK	2012-2016	460	46.6	26.9	24.3	1.3	176/460 (38.6)	NR	NR	NR	NR	NR
Bellettini	2022	Retrospective MC	Città della Salute e della Scienza Hospital, Torino; Sant'Orsola-Malpighi Hospital, Bologna; Italy	2000-2018	657	53	22.2	4.3	0,04	67/657 (10.2)	NR	NR	38/657 (5.8)	NR	NR
Benck	2021	Retrospective SC	Cedars Sinai Medical Center, USA	2012-2018	367	NR	NR	NR	NR	NR	NR	NR	NR	NR	NR
Carey	2023	Retrospective SC	Presbyterian Hospital, New York, USA	2010-2019	490	53	26.3	56.1	4.3	NR	NR	NR	75/490 (15.3)	NR	NR
Chinnadurai	2022	Retrospective SC	Montefiori Medical Center, USA	2006-2017	243	53.6	29.6	62.3	0.4	NR	NR	NR	21/243 (8.6)	NR	NR
Coutance	2019	Retrospective SC	Pitié Salpêtrière Hospital, France	2009-2015	412	NR	38.6	NR	NR	NR	NR	NR	138/412 (33.5)	NR	NR
Giangreco	2021	Prospective and retrospective MC	Columbia University Irving Medical Center, New York, USA; Cedars-Sinai Hospital, California, USA; Pitié Salpêtrière University Hospital, Paris, France	2014-2016	88	56.7	30.7	23.7	NR	42/88 (47.7)	NR	NR	42/88 (47.7)	NR	NR
Gong	2018	Retrospective SC	Baylor University Medical Center, Dallas, USA	2012-2016	253	58.8	23.3	26.5	2	64/253 (25.3)	31/253 (12.3)	15/253 (5.9)	18/253 (7.1)	33/253 (13)	NR
Gosling	2023	Retrospective SC	Duke University Medical Center, Durham, USA	2009-2019	461	56.1	31.7	33	NR	195/461 (42.2)	NR	126/461 (27.3)	69/461 (15.0)	NR	NR
Han	2024	Retrospective MC	Stanford University, Cedars Sinai medical center, USA	2015-2019	596	55.4	27.6	23.3	0.9	NR	NR	NR	NR	NR	NR
Hoemann	2020	Retrospective SC	Columbia University Medical Center	2010-2017	381	54.1	25.7	60.4	NR	NR	NR	NR	53/381 (13.9)	NR	NR
Jamil	2017	Retrospective SC	Baylor University Medical Center, Dallas, USA	2012-2016	255	58.3	23.9	32.2	3	NR	NR	NR	NR	34/255 (13.3)	NR
Jenryd	2022	Prospective SC	Skane University Hospital, Lund, Sweden	2015-2018	63	51.2	30.2	44.4	NR	NR	NR	NR	8/63 (12.7)	NR	NR
Kransdorf	2023	Retrospective SC	Cedars Sinai Medical Center, USA; Stanford University Medical Center, USA	2012-2019	1079	56.8	27.8	29.8	NR	222/1079 (20.6)	NR	NR	63/1079 (5.8)	NR	NR
Kuzemchak	2021	Retrospective SC	Vanderbilt University Medical Center, Tennessee, USA	2012-2019	201	52.15	24.9	52.7	NR	132/201 (65.7)	NR	117/201 (58.2)	15/201 (7.5)	NR	NR
Lozano-Edo	2021	Prospective SC	La Fe Hospital, Valencia, Spain	2009-2019	135	53.0	20	27.4	20	37/135 (27.4)	NR	NR	19/135 (14.1)	NR	10/135 (7.4)
Moayedi	2024	Retrospective MC	USA	2010-2020	2746	53.8	25.2	33.3	1.5	NR	NR	NR	215/2746 (7.8)	NR	NR
Nagy	2020	Retrospective SC	Semmelweis University, Budapest, Hungary	2015-2019	151	53.5	26.5	16.6	NR	29/151 (19.2)	3/151 (2)	3/151 (2.0)	19/151 (12.6)	NR	4/151 (2.6)
Nagy	2021	Retrospective SC	Semmelweis University, Budapest, Hungary	2012-2018	297	54.0	25.9	NR	NR	56/296 (18.9)	NR	NR	43/297 (14.5)	NR	NR
Nakamura	2020	Retrospective SC	Osaka University, Osaka, Japan	2007-2016	69	39.2	31.9	92.8	NR	12/69 (17.4)	NR	5/69 (7.2)	4/69 (5.8)	NR	3/69 (4.3)
Nicoroa	2017	Retrospective SC	Duke University Medical Center, Durham, USA	2009-2014	317	58.0	25.9	32.9	N/A	99/317 (31.2)	NR	NR	39/317 (12.4)	NR	17/317 (5.4)
Palani	2021	Retrospective SC	India	2010-2019	100	41.4	21	1	1	40/100 (40)	NR	NR	28/100 (28)	NR	7/100 (7)
Peled	2020	Retrospective SC	Sheba Medical Center, Israel	1997-2017	275	50.6	16.7	17.1	NR	100/275 (36.4)	60/275 (21.8)	19/275 (6.9)	1/275 (0.4)	20/275 (7.3)	20/275 (7.3)
Peled	2020	Retrospective SC	Sheba Medical Center, Israel	1995-2018	209	49.6	17.7	29.4	NR	80/209 (38.3)	41/209 (19.6)	14/209 (6.7)	3/209 (1.4)	NR	22/209 (10.5)
Peled	2018	Prospective SC	Sheba Medical Center, Israel	1990-2017	168	48.6	19.6	17.3	NR	72/168 (42.9)	NR	NR	NR	NR	NR
Prieto	2018	Prospective SC	Coimbra Hospital, Coimbra, Portugal	2003-2015	290	54.5	22.8	NR	3.1	47/290 (16.2)	15/290 (5.2)	17/290 (5.9)	15/290 (5.2)	NR	14/290 (4.8)
Quintana-Quezada	2016	Retrospective SC	Memorial Hermann Hospital, Texas	2012-2015	99	NR	NR	NR	NR	18/99 (18.2)	3/99 (3)	2/99 (2)	9/99 (9.1)	NR	4/99 (4.0)
Ram	2023	Retrospective SC	Sheba Medical Center, Israel	1999-2019	175	48.6	0,2	0,24	NR	71/175 (40.6)	37/175 (21.1)	12/175 (6.9)	3/175 (1.75)	NR	19/175 (10.9)
Rega	2024	RCT, MC	Europe and UK	2020-2023	204	57	22	29	NR	50/204 (24.5)	NR	11/204 (5.4)	26/204 (13.7)	NR	13/204 (6.4)
Rhee	2021	Retrospective SC	Asan Medical Center, Seoul, South Korea	1992-2017	570	46.7	28.9	NR	9.1	38/570 (6.7)	1/570 (0.2)	14/570 (2.5)	20/570 (3.5)	34/570 (6.0)	3/570 (0.5)
Sabatino	2017	Retrospective MC	University of Bologna, Italy; ISMETT, Palermo, Italy	1999-2013	518	52.2	19	1	5	72/518 (13.9)	4/518 (0.8)	33/518 (6.4)	35/518 (6.8)	NR	NR
Servais	2024	Retrospective SC	Nebraska, USA	2012-2018	200	NR	71	70	NR	NR	NR	NR	20/200 (10)	NR	NR
Smith	2021	Retrospective SC	University of Pittsburgh Medical Center, USA	2005-2017	448	55.0	23.4	36.4	NR	43/448 (16.5)	10/448 (2.2)	5/448 (1.1)	35/448 (7.8)	NR	24/448 (5.4)
Squiers	2017	Retrospective SC	Baylor University Medical Center, Texas, USA	2012-2015	191	57	25	47.1	1.6	59/191 (30)	35/191 (18.3)	8/191 (4.2)	16/191 (8.4)	24/191 (12.6)	NR
Still	2018	Retrospective SC	Baylor University Medical Center, Texas, USA	2012-2016	255	59.0	24	31	2	65/255 (25.5)	31/255 (12.1)	15/255 (5.9)	19/255 (7.5)	NR	NR
Takahasi	2020	Retrospective SC	Barnes-Jewish Hospital, Missouri, USA	2011-2017	100	53.8	27	71	1	17/100 (17)	NR	8/100 (8)	9/100 (9)	17/100 (17)	0
Truby	2021	Retrospective SC	Duke University Medical Center, USA	2016-2020	131	52.0	32.3	29	NR	39/131 (29.8)	NR	24/131 (18.3)	15/131 (11.5)	39/131 (29.8)	NR
Wright	2017	Retrospective SC	Columbia University Medical Center, USA	2010-2014	269	53.9	25.7	60.6	NR	NR	NR	NR	29/269 (10.8)	NR	NR

ECMO, extracorporeal membrane oxygenation; LVAD, left ventricular assist device; MC, multi-center; NR: not reported; PGD, primary graft dysfunction; RV, right ventricle; RCT, randomized, controlled trial; SC, single-center; UK, United Kingdom; USA, United States of America

Of the 39 included studies, 33 were retrospective in nature (85%), and 1 study was a randomized, controlled trial. Total participants ranged from 63 to 2746 patients, incidence of PGD ranged from 6.7% to 65.7%. A total of 11 studies (28%) did not specify total incidence of PGD in their respective cohorts, 12 studies (31%) classified patients into all corresponding categories of LV-PGD severity according to the ISHLT consensus definition. The incidence of RV-PGD was specified in 13 studies, while no study assessed risk factors for RV-PGD.

There was notable heterogeneity among included studies regarding composition of the study cohort, as reflected by the variable age, percentage of female patients, recipient durable LVAD prior to HTX, definition of assessed risk factors, and reporting of PGD severity. All PGD was reported as primary outcome in 20 studies (51%), while severe PGD as primary outcome was recorded in 14 studies (36%). A total of 7 studies reported a composite endpoint of moderate-severe PGD (18%). Three studies assessed both all PGD and severe PGD as outcome, while another did the same for all PGD and moderate-severe PGD. No study included cardiac donation after circulatory death (DCD) in their analysis.

### Quality of included studies

Included studies were of varying quality. A total of 24 studies scored a high risk of bias on the domain of Confounding Factors. This was mostly because of the use of an inadequate threshold used during univariate analysis (*p*<0.2) for selection of prognostic factors for multivariate analysis. Furthermore, a total of 25 studies scored moderate to high risk of bias in the domain Statistical Analysis & Reporting, which was mainly due to selective reporting of (significant) data from multivariate analysis. 19 studies scored moderate to high risk in Study Participation, mainly as a result of cohort selection. The included randomized, controlled trial scored an overall low risk of bias. Results of quality assessment can be found in the supplementary files (Table S1).

### Risk factors for all PGD

**Figure 2:**
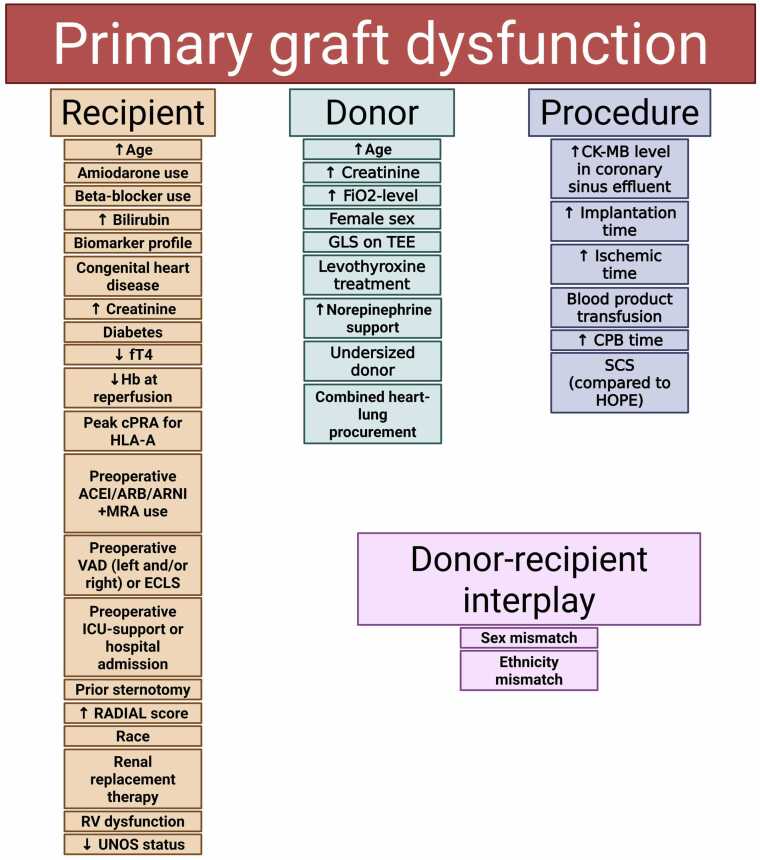
All identified risk factors. ACEi, angiotensin-converting enzyme inhibitor; ARB, antiogensin receptor blocker; ARNI, angiotensin-receptor neprilysin inhibitor; CPB, cardiopulmonary bypass; cPRA, calculated panel reactive antibodies; ECLS, extracorporeal life support; fT4, free T4; FiO2, fraction of inspired oxygen; GLS, global longitudinal strain; Hb, hemoglobin level; HLA-A, human leukocyte antigen A; ICU, intensive care unit; MRA, mineralocorticoid receptor antagonist; RV, right ventricle; SCS, static cold storage; TEE, transesophageal echocardiography; VAD, ventricular assist device; UNOS, United Network for Organ Sharing. HOPE, hypothermic, oxygenated perfusion. The image was created using Biorender.com

In all studies, a total of 37 risk factors for all PGD were identified by multivariate analysis. These factors were categorized into recipient-related (20), donor-related (9), and procedure-related (6) risk factors, with an additional 2 involving an interplay between donor and recipient (Figure 2).

The recipient-related risk factors included incremental age, amiodarone therapy, beta-blocker use, increased bilirubin levels, a specific biomarker profile, a history of congenital heart disease, increased creatinine levels, diabetes, decreased free T4 levels, decreased hemoglobin level at reperfusion of the transplanted heart, peak calculated panel reactive antibodies for human leukocyte antigen A, preoperative ace-inhibitor/angiotensin-receptor blocker/angiotensin-receptor neprilysin-inhibitor and mineralocorticoid receptor antagonist use, preoperative ventricular assist device or extracorporeal life support therapy, preoperative intensive care support or hospital admission, prior sternotomy (other than for LVAD implantation), increased RADIAL scores, African-American race, renal-replacement therapy at time of transplant, right ventricular dysfunction as calculated by pulmonary artery pulsatility index, and a lower UNOS status indicating higher urgency. The donor-related risk factors were incremental age, increased creatinine levels, increased fraction of inspired oxygen, female gender, global longitudinal strain on transesophageal echocardiography, levothyroxine treatment, incremental norepinephrine support, an undersized donor, and combined heart-lung procurement. Procedure-related risk factors were incremental CK-MB levels in the coronary sinus effluent, incremental implantation time, incremental ischemic time, blood product transfusion, incremental cardiopulmonary bypass time, and the use of static cold storage as preservation method when compared to hypothermic oxygenated perfusion. The factors on the donor-recipient interplay were a sex and ethnicity mismatch. The recipient biomarker profile included serum, peroxiderin 2, tropomyosin alpha-4, C-type lectin domain family 4 and myeloperoxidase, all of which were associated with an increased risk of severe PGD.^12^, ^31^ An overview of all assessed and identified risk factors (both significant and non-significant) in the original studies can be found in the supplementary files (Table S2).

### Risk factors for severe PGD

A total of 15 studies identified 26 risk factors for severe PGD. The incidence of severe PGD in the combined cohorts was 11% (719/6664 patients). Identified risk factors for severe PGD are listed in Table 3.

**Table 3:** Risk Factors for Severe Primary Graft Dysfunction

Risk factors for severe PGD		
Recipient	Donor	Procedure
ACEi/ARB/ARNI+MRA treatment	Female donor	Blood product transfusion (RBC and platelets)
Amiodarone treatment	Higher age	Cardiopulmonary bypass time
Amiodarone + BB therapy	Increased FiO2	CK-MB ≥11 ng/ml in coronary sinus effluent
Congenital heart disease	Increased creatinine level	Female-male mismatch
Diabetes mellitus	Norepinephrine >3 mg/h	Implantation time
Female recipient	Undersized donor	Increased cold ischemic time
LVAD		Static cold storage (compared to HOPE) in DBD transplantation
Pretransplant anemia		
Pretransplant PVR >3WU		
Primary clotting/bleeding disorder		
Prior sternotomy (other than LVAD)		
Renal-replacement therapy		
Serum biomarker profile (tropomyosin alpha-4, myeloperoxidse, peroxiderin 2)		

ACEi, angiotensin-converting enzyme inhibitor; ARB, angiotensin-receptor blocker; ARNI, angiotensin-receptor/neprilysin inhibitor; BB, beta-blocker; CK-MB, creatinine kinase MB; DBD, donation after brain death; FiO2, fraction of inspired oxygen; HOPE, hypothermic oxygenated perfusion; LVAD, left ventricular assist device; MRA, mineralocorticoid receptor antagonist; ng; nanogram; PVR, pulmonary vascular resistance; RBC, red blood cell; WU, woods units

### Meta-analysis of risk factors for severe PGD

**Figure 3:**
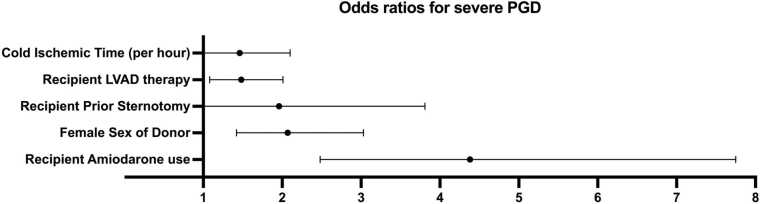
Results of meta-analysis for risk factors of severe primary graft dysfunction. LVAD, left ventricular assist device

A total of 6 risk factors were identified in ≥2 studies that could be analyzed using meta-analysis (Figure 3). These include recipient prior sternotomy (other than for LVAD implantation) (2 studies), recipient LVAD prior to HTX (6 studies), recipient amiodarone therapy (3 studies), female sex of the donor (2 studies), cold ischemic time per hour increment (5 studies) and perioperative blood product (red blood cells, platelets) administration (3 studies). Of these, recipient amiodarone therapy is the strongest predictor for severe PGD (OR: 4.38; 95% CI: 2.48-7.75), followed by female sex of the donor (OR: 2.07; 95% CI: 1.42-3.03), recipient prior sternotomy (other than for LVAD implantation) (OR: 1.96; 95% CI: 1.01-3.81), recipient LVAD therapy (OR: 1.48; 95% CI 1.08-2.01) and cold ischemic time per hour increment (OR: 1.46; 95% CI 1.01-2.10). Perioperative blood product administration was excluded from meta-analysis due to heterogeneity in definition (per unit, per 100 ml and per 1000 ml) but is highlighted here due to its significance as a predictor for severe PGD in all identified studies. Forest plots of all individual meta-analyses can be found in the supplementary files (Figure S1-S6).

## Discussion

**Figure 4:**
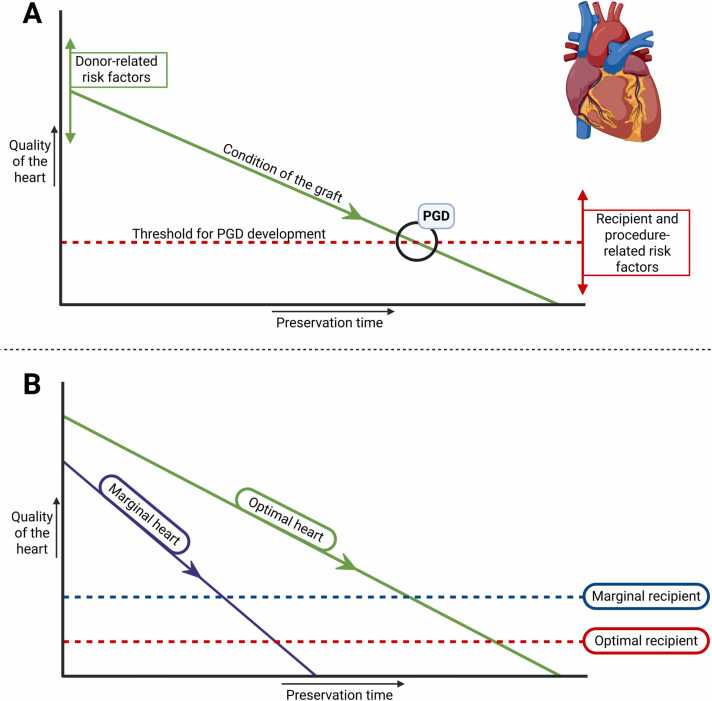
Schematic representation of the proposed interplay between donor-, procedure-, and recipient-related risk factors for primary graft dysfunction (PGD). A. The green line represents the condition of the donor organ. The starting point of the green line is primarily determined by donor-related factors, which determines the quality of the graft. The slope of the line represents the decline in graft condition over the course of preservation. The interrupted red line represents the threshold at which PGD develops, which is determined by recipient and procedure-related risk factors. PGD develops when the green and red line intercept. B. Example of scenario’s in which either the donor heart (for instance, due to older donor age and the presence of left ventricular hypertrophy), or the recipient (for instance due to prior left ventricular assist device implantation and chronic amiodarone use) is of marginal status. Procedure-related risks factors (for instance prolonged cardiopulmonary bypass time and the requirement for blood product transfusion) act as effect modifiers. The image was created using Biorender.com

We conducted the first systematic review and meta-analysis since the introduction of the ISHLT consensus definition on PGD in 2014 which specifically focuses on identifying risk factors. By conducting a systematic search of the literature, we were able to identify 37 risk factors for PGD after HTX, of which 26 where specifically associated with severe PGD Despite notable heterogeneity among studies, 5 risk factors associated with severe PGD could be identified by meta-analysis, while blood product administration was also identified in multiple studies as an important risk factor for severe PGD. This is supplemented on top of a prior systematic review by Buchan et al.,^5^ focusing primarily on incidence of PGD, who conducted additional meta-analysis to identify risk factors for PGD isolated from 15 studies. While partially overlapping, our study focused exclusively on the identification of risk factors and hence we were able to identify an additional 24 studies to include in our systematic review. We therefore believe our study is the most complete overview of known risk factors for PGD to date. Furthermore, while the meta-analysis of Buchan et al. focused on risk factors for all PGD, we specifically focused on the risk factors for severe PGD, explaining some of the differences found. Lastly, our study includes results from a recent randomized, controlled trial that identified preservation of hearts using static cold storage as a significant risk factor for the occurrence of severe PGD, when compared to preservation using hypothermic, oxygenated perfusion.^46^ Whilst only identified in a single study (and hence not included in meta-analysis), the robust level of evidence suggests that the use of hypothermic, oxygenated perfusion has a true protective effect on the occurrence of severe PGD when compared to static cold storage in a context of donation after brain death. Although the exact pathophysiology of PGD is still unknown, cumulating evidence suggests an interplay between donor, recipient, and procedure-related factors that contribute to a decreased threshold for PGD development. These factors include ischemia and subsequent reperfusion injury, but also pathophysiology of donor death and prior health status, mode of preservation, and health status of the recipient.^47^, ^48^ The interaction between each individual risk factor most likely determines the risk and severity of PGD, more so than the presence of a single risk factor (Figure 4).

The occurrence of PGD is an important predictor for early mortality after HTx. Especially severe PGD, which by definition encompasses dependence on veno-arterial extracorporeal life support (VA-ECLS), invasive left, or biventricular support for survival,^6^ greatly affects outcome. Traditionally, VA-ECLS has been associated with a great impact on patient morbidity. A recent meta-analysis^49^ revealed that the need for VA-ECLS after HTX could be associated with 33% in-hospital mortality at 30 days and 50% mortality at 1 year in hearts donated after brain death. The same study revealed that these patients are also at additional risk of developing complications associated with VA-ECLS, including bleeding (38%), infection (21%), limb-ischemia (5%), and stroke (4%). Thus, preventing severe PGD (and hence the need for VA-ECLS) after HTX might be crucial for improving outcomes, and awareness of risk factors for PGD could contribute to this. An example of this is improving donor-recipient matching. The use of marginal donor hearts is associated with decreased 1- and 5-year survival rates,^50^, ^51^ hence avoiding matching a marginal donor heart with a recipient with known risk factors for severe PGD may improve outcomes. Moreover, individual risk factors could be addressed by utilizing machine perfusion to limit cold ischemic time^46^ or by reducing amiodarone dose when a patient is listed for HTX, as was described in a small pilot study.^40^ Additionally, knowledge of risk factors for severe PGD could aid in identifying recipients who may benefit from proactive monitoring and early supportive therapy that allows the newly transplanted heart to recover, thereby preventing the need for VA-ECLS. Examples of this include meticulous perioperative fluid management, extensive reperfusion of the graft in the recipient while on cardiopulmonary bypass, or temporary circulatory support aimed at ventricular unloading. Especially the latter might be an interesting intervention by proactively initiating less invasive options for ventricular support, such as an intra-aortic balloon pump or Impella (Abiomed, Danvers, MA, USA) that have a more favorable complication and risk factor profile than VA-ECLS,^52^, ^53^ although no head-to-head comparison in case of HTx has been conducted. By timely intervention, the downward spiral of low output syndrome combined with side-effects of escalating inotropic support^54^ (e.g., decreased systemic vascular resistance, arrhythmia’s, intracellular calcium overload, mesenteric ischemia, acute kidney injury, etc.) resulting in progressive hemodynamic compromise and end-organ dysfunction necessitating VA-ECLS may be prevented. Interestingly, other studies suggest that technological advancements have significantly reduced the major complications associated with VA-ECLS in this cohort of patients, facilitating direct and early escalation to VA-ECLS.^55^ A historical cohort study from Columbia University recently found that early escalation to VA-ECLS might be associated with a decreased mortality rate as opposed to a conservative strategy (5% vs 28%, respectively).^56^ When confirmed in future studies, this would be an advocate for safe and early institution of VA-ECLS after transplantation.

Prior to establishment of the ISHLT consensus definition on PGD, the RADIAL score was introduced to estimate the risk of primary graft failure after HTX^57^ and was subsequently validated.^58^ Although the original paper was excluded from this review due to publication prior to establishment of the ISHLT consensus definition in 2014, it’s interesting to note that the RADIAL score seems to have survived the test of time and could still serve as a valuable tool in identifying patients at increased risk for PGD. This is supported by our findings, which identify the RADIAL score as a separate risk factor for PGD^20^ and the identification of multiple studies that seem to confirm the risk factors constituting the original RADIAL score as risk factors for ISHLT-defined PGD (i.e., recipient and donor age, diabetes, inotrope support, length of ischemia). On the other hand, the RADIAL score did not seem to possess major predictive power for contemporary PGD in a recent cohort study.^9^ Based on this, the RADIAL score might still be a valuable tool for quick risk assessment in specific settings, although its predictive powers should not be overestimated.

The heterogeneity observed among the included studies is a critical finding of our systematic review. The primary sources of heterogeneity were cohort composition, the definition of assessed risk factors, and outcome definitions. The variability in defining risk factors was mainly due to different cut-off points, the definition of separate categories based on continuous variables, or assessing OR’s based on uncategorized continuous variables. An example of this is total ischemic time, which was classified as total ischemic time per minute-increment, per 10-minute-increment, per hour-increment and >240 min. Although transformation of data would be possible for meta-analysis, we refrained from this as it would risk introducing additional bias into the data with every transformative step. Furthermore, while all studies adhered to the ISHLT consensus definition of PGD, the severity of PGD used as primary outcome varied. This complicates the assessment of each individual risk factor in relationship to the occurrence of PGD. Additional limitations observed in the literature include selective reporting of (significant) risk factors for severe PGD after multivariate analysis, which could have skewed results by introducing publication bias. Additional bias could also have been introduced by the retrospective nature of most included studies (Table 2). Given the above, we recommend that additional evidence from future studies is needed, with repetition of meta-analysis in the coming years to confirm and solidify identified risk factors for PGD.

In addition to the aforementioned considerations, future studies should focus on identifying risk factors for PGD-RV and investigate the influence of cardiac DCD on incidence of PGD. In the current literature, most attention is focused on the development of PGD-LV with limited attention paid to PGD-RV or none at all. Only one recent study^59^ specifically addressed the occurrence of right ventricular failure after HTX, which was not included in our review due to a modified outcome definition not meeting our inclusion criteria. They reported a 59% incidence of right ventricular dysfunction and a 9% incidence of severe right ventricular dysfunction in a cohort of HTX-recipients. Reported 30-day mortality of severe right ventricular dysfunction was 60%, the need for renal-replacement therapy approached 50% after 15 days in this subgroup. Notably, identified risk factors for severe right ventricular dysfunction differed somewhat from those for severe PGD-LV, including female recipient sex and elevated bilirubin levels, but not ischemic time, prior LVAD, or amiodarone therapy. This suggests that the pathogenesis of severe right ventricular dysfunction might differ from that of severe PGD-LV after HTX, emphasizing the need for further research. Moreover, the introduction of cardiac DCD has added a whole new subset of donors, the influence of which on PGD incidence is not yet fully elucidated. Evidence currently suggests that cardiac DCD is associated with a higher incidence of severe PGD,^60^, ^61^, ^62^, ^63^ likely due to the impact of warm ischemia and right ventricular distention on graft quality.^64^, ^65^ However, such evidence has not been sufficiently demonstrated and may be confounded by several factors, including those related to the absence of brainstem death, the use of machine perfusion, and pathophysiological changes in the donor associated with circulatory death. Interestingly, some evidence suggests that recovery from severe PGD is associated with better outcomes in DCD recipients than in conventional recipients, with shorter duration of invasive ventricular support and hospital stay.^61^ This suggests the existence of unrecognized underlying differences between both donor groups and underscores the need for additional research into this topic.

Lastly, since the publication of the 2014 ISHLT consensus definition of PGD, the field of HTX has seen tremendous advances in terms of graft preservation technology (e.g., the influence and emergence of novel normothermic and hypothermic preservation strategies) and clinical knowledge of the etiology and pathophysiology of PGD (e.g., the identification of a second, “delayed” clinical trajectory related to vasoplegia^66^). Due to the abovementioned developments and the recognition that distinction between “primary” and “secondary” causes might not always be clear, a revision of the current consensus definition of PGD by the ISHLT seems justified.^67^

In conclusion, we have conducted the first comprehensive systematic review specifically aimed at identifying risk factors for PGD according to the ISHLT consensus definition since it was established in 2014. We identified 37 risk factors for PGD and solidified 5 of them for severe PGD by meta-analysis. These findings could serve as guidance for clinicians and future studies.

## Author contributions

M.V., S.K.g.D., and J.K. conducted the search, screening, and study selection. M.V. drafted the manuscript and incorporated feedback. S.K.g.D., E.B., M.M., S.B., M.O., and N.v.d.K. critically revised the manuscript for intellectual content. All authors provided final approval of the submitted version.

## Funding

This paper is supported by the partners of Regenerative Medicine Crossing Borders (RegMed XB), a public-private partnership that uses regenerative medicine strategies to cure common chronic diseases. This collaboration project is financed by the Dutch Heart Foundation and the Dutch Ministry of Economic Affairs by means of the public-private partnership allowance made available by the Top Sector Life Sciences & Health to stimulate public-private partnerships.

## Disclosure statement

The authors declare no conflict of interest.

## Classification

systematic review & meta-analysis.

## Declaration of Competing Interest

The authors declare that they have no known competing financial interests or personal relationships that could have appeared to influence the work reported in this paper.

## Footnotes

Appendix A Supplementary data associated with this article can be found in the online version at doi:10.1016/j.jhlto.2026.100483.
