# Supplementary material for: Risk factors for primary graft dysfunction after heart transplantation—a systematic review and meta-analysis
Source: JHLT Open. 2026 Jan 30;12:100483. doi: 10.1016/j.jhlto.2026.100483 (PMC13019779; doi:10.1016/j.jhlto.2026.100483)

**Supplementary files.**

**Appendix**

*S1: search string*

PubMed:

("heart transplant*"[Title/Abstract] OR "cardiac transplant*"[Title/Abstract] OR "heart graft*"[Title/Abstract] OR “cardiac graft*”[Title/Abstract] OR "heart transplantation"[MeSH Terms]) AND ("primary graft dysfunction"[Title/Abstract] OR "primary graft failure"[Title/Abstract] OR "graft dysfunction"[Title/Abstract] OR "graft failure"[Title/Abstract] OR "PGD"[Title/Abstract] OR “Early graft failure”[Title/Abstract] OR “Early graft dysfunction”[Title/Abstract] OR "primary graft dysfunction"[MeSH Terms])

Embase:

('heart transplant*':ti,ab,kw OR 'cardiac transplant*':ti,ab,kw OR 'heart graft*':ti,ab,kw OR ‘cardiac graft*’:ti,ab,kw OR 'heart transplantation'/exp) AND ('primary graft dysfunction':ti,ab,kw OR 'primary graft failure':ti,ab,kw OR 'graft dysfunction':ti,ab,kw OR 'graft failure':ti,ab,kw OR 'PGD':ti,ab,kw OR ‘early graft failure’:ti,ab,kw OR ‘early graft dysfunction’:ti,ab,kw OR 'primary graft dysfunction'/exp)

Cochrane:

(primary graft dysfunction):ti,ab,kw AND (heart transplantation):ti,ab,kw

**Tables**

S1. Results of quality assessment using both Quality in Prognosis Studies tool (QUIPS) and the Risk of Bias 2 (RoB2). D1: randomization; D2: effect of assignment and adherence to intervention; D3: missing outcome data; D4: measurement of outcome; D5: selection of reported results.

S2: all assessed risk factors, both significant and non-significant.

| **Author** | **Year** | **Recipient risk factor** | **Odds ratio (95% CI)** | **Donor risk factor** | **Odds ratio (95% CI)** | **Procedural risk factor** | **Odds ratio (95% CI)** |
| --- | --- | --- | --- | --- | --- | --- | --- |
| Avtaar Singh | 2019 | All PGD: Recipient diabetes | 2.04 (0.9993-4.1720) | All PGD: Donor age | 1.02 (1.0043-1.0383) | All PGD: Implant time per incremental minute | 1.01 (1.0005-1.0195) |
|  |  | ALL PGD: Preoperative VAD/ECMO | 1.79 (1.1371-2.8295) |  |  | All PGD: Female-male mismatch | 1.74 (1.0465-2.9086) |
|  |  | Severe PGD: recipient sternotomy | 3.21 (1.3215-7.8084) |  |  | Severe PGD: Implant time | 1.02 (1.0003-1.0342) |
|  |  |  |  |  |  | Severe PGD: Female-male mismatch | 2.43 (1.0966-5.3722) |
| Avtaar Singh | 2019 | All PGD: recipient diabetes | 3.04 (149-6.21) | All PGD: donor age | <21: reference range | All PGD: Implant time | <45: reference range |
|  |  | All PGD: recipient MCS | 2.73 (1.35-5.55) |  | 21-40: 1.44 (0.72-2.85) |  | 46-60: 1.8 (1.11-2.93) |
|  |  |  |  |  | 41-50: 1.81 (0.89-3.67) |  | 61-90: 1.96 (1.22-3.15) |
|  |  |  |  |  | >50: 2.53 (1.26-5.37) |  | >90: 2.15 (1.01-4.60) |
|  |  |  |  |  |  | All PGD: Bypass time >180 min | 2.53 (1.75-3.66) |
| Bellettini | 2022 | All PGD: right ventricular dysfunction (PAPi <1.68) | 1.7 (1.02-3.3) |  |  |  |  |
| Benck | 2021 | Severe PGD: prior cardiac surgery | 1.55 (NR-NR) |  |  | Severe PGD: ischemic time (per hour) | 1.98 (NR-NR) |
|  |  | Severe PGD: ACEi/ARB/ARNI + MRA | 2.41 (NR-NR) |  |  | Severe PGD: transfusion of PRBC (per unit) | 1.14 (NR-NR) |
|  |  | Severe PGD: AMIO + BB | 5.47 (NR-NR) |  |  | Severe PGD: transfusion of platetlets (per unit) | 1.14 (NR-NR) |
| Carey | 2023 | Severe PGD: primary clotting/bleeding disorder | 3.15 (1.01-9.86) |  |  |  |  |
| Chinnadurai | 2022 | Severe PGD: recipient MCS | 5.18 (1.08-24.85) |  |  |  |  |
|  |  | Per year-increment | 1.81 (1.16-2.81) |  |  |  |  |
|  |  | Severe PGD: amiodarone use | 3.37 (1.22-9.29) |  |  |  |  |
|  |  | Per 100mg preoperative dose | 1.35 (1.05-1.71) |  |  |  |  |
|  |  | Severe PGD: Recipient diabetes | 4.90 (1.60-15.00) |  |  |  |  |
|  |  | Severe PGD: intraoperative RBC transfusion per 100ml-increment | 1.08 (1.02-1.14) |  |  |  |  |
|  |  | Severe PGD: Hypertension | 0.16 (0.05-0.49) |  |  |  |  |
|  |  | Severe PGD: duration of MCS support (per year-increment) + amiodarone (per 100mg increment) | 1.43 (1.15-1.78) |  |  |  |  |
| Coutance | 2019 |  |  | Severe PGD: female donor | 1.88 (1.21-2.94) |  |  |
|  |  |  |  | Severe PGD: donor norepinephrine use > 3mg/h | 3.01 (1.69-5.37) | Severe PGD: cold ischemic time (per hour) | 1.66 (1.31-2.09) |
| Giangreco | 2021 | Severe PGD: inotrope therapy | 0.4342 (0.3043-0.6033) |  |  |  |  |
|  |  | Severe PGD: MCS therapy | 1.192 (1.000-1.781 |  |  |  |  |
|  |  | Severe PGD: Plasma Kallikrein | 0.1959 (0.0592-0.3663) |  |  |  |  |
|  |  | Severe PGD: Plasma peroxeridoxin 2 | 10.7457 (3.7618-29.2799) |  |  |  |  |
|  |  | Severe PGD: Plasma tropomyosin alpha-4 | 10.8144 (4.5053-22.427) |  |  |  |  |
|  |  | Severe PGD: plasma myeloperoxidase | 7.685 (2.7906-15.6071) |  |  |  |  |
| Gong | 2018 | Moderate/severe PGD: preoperative creatinine (continuous scale) | 3.15 (1.17-8.43) | Moderate/severe PGD: donor undersized ≥30% by predicted heart mass | (3.27 (1.25-8.58) |  |  |
| Gosling | 2023 | All PGD: recipient T3 | 1.25 (0.8-1.95) |  |  |  |  |
|  |  | Severe PGD | 0.77 (0.39-1.48) |  |  |  |  |
| Han | 2024 | All PGD: beta-blocker use | 2.16 (1.31-3.60) | All PGD: donor age | 1.02 (1.00-1.04) | All PGD: total ischemic time (min) | 0.997 (0.994-0.999 |
|  |  | All PGD: prior cardiac surgery | 1.52 (1.00-2.32) |  |  |  |  |
|  |  | All PGD: Inotrope use before transplant | 0.76 (0.47-1.22) |  |  |  |  |
|  |  | All PGD: UNOS status 1 or 2 | 1.92 (1.10-3.34) |  |  |  |  |
|  |  | All PGD: UNOS status 4-6 | 1.45 (0.88-2.40) |  |  |  |  |
|  |  | All PGD: Peak cPRA for HLA-A | 4.26 (1.44-12.6) |  |  |  |  |
| Hoemann | 2020 | Severe PGD: prior VAD | 1.43 (0.63-3.22) |  |  | Severe PGD: PRBC administration (per unit) | 3.16 (2.18-4.57) |
|  |  | Severe PGD: amiodarone treatment <6 months prior to HTx, continued to HTx | 3.70 (1.26-10.88) |  |  |  |  |
|  |  | Severe PGD: amiodarone treatment <6 months prior to HTx, discontinued to HTx | 0.416 (0.08-2.15) |  |  |  |  |
| Jamil | 2017 | Moderate/severe PGD: recipient age ≥65 years | 0.32 (0.11-0.98) | Moderate/severe PGD: donor undersized by predicted heart mass (continuous scale) | 1.03 (1.00-1.05) |  |  |
|  |  | Moderate/severe PGD: creatinine ≥2.0 mg/dL | 3.46 (1.19-10.1) |  |  |  |  |
| Jenryd | 2022 |  |  | Severe PGD: CK-MB ≥11 ng/ml in coronary sinus after preservation | 7.4 (1.13-48.46) |  |  |
| Kransdorf | 2023 | Severe PGD: amiodarone + BB | 1.35 (1.20-1.5) | Severe PGD: Age (per 10 years) | 1.11 (1.08-1.15) | Severe PGD: Predicted Heart Mass ratio (per 10%) | 0.84 (0.8-0.9) |
|  |  | Severe PGD: RVAD/BiVAD/TAH | 1.82 (1.2-2.15) | Severe PGD: FiO2 (per 10%) | 1.08 (1.06-1.09) |  |  |
|  |  |  |  | Severe PGD: Log Creatinine | 1.43 (1.25-1.5) |  |  |
| Kuzemchak | 2021 | All PGD: female recipient | 0.577 (0.280-1.191) |  |  | All PGD: WIT | 0.997 (0.979-1.016) |
|  |  | All PGD: preoperative ICU-support | 3.089 (1.351-7.061) |  |  | All PGD: TIT | 1.004 (0.999-1.010) |
|  |  |  |  |  |  | All PGD: CPB (min) | 1.012 (1.001-1.022) |
|  |  |  |  |  |  | All PGD: Transfusions | 1.418 (0.887-2.264) |
| Lozano-Edo | 2021 | All PGD: serum SERCA2a ≥0.6 ng/mL | 0.215 (0.075-0.614) |  |  |  |  |
|  |  | All PGD: bilirubin level | 1.849 (1.152-2.969 |  |  |  |  |
|  |  | All PGD: preoperative MCS BTT | 3.762 (1.280-11.052) |  |  |  |  |
| Moayedi | 2024 | Severe PGD: renal replacement therapy | 2.41 (1.31-4.43 |  |  | Severe PGD: total ischemic time per hour | 1.20 (1.02-1.41 |
|  |  | Severe PGD: preoperative MCS | 1.77 (1.13-2.77) |  |  |  |  |
| Nagy | 2020 | All PGD: UNOS summary score | 1.3 (1.13-1.5) | All PGD: preoperative levothyroxine treatment | 1.07 (0.21-5.41) |  |  |
|  |  | All PGD: hypothyroidism | 0.51 (0.09-2.95) |  |  |  |  |
|  |  | All PGD: hyperthyroidism | 0.61 (0.11-3.34) |  |  |  |  |
|  |  | All PGD: preoperative levothyroxine treatment | 2.04 (0.84-4.94) |  |  |  |  |
|  |  | All PGD: amiodarone treatment | 1.3 (0.55-3.06) |  |  |  |  |
|  |  | All PGD: low fT4 | 6.49 (2.26-18.6) |  |  |  |  |
| Nagy | 2021 |  |  | All PGD: methylprednisolone treatment | 0.38 (0.16-0.90) |  |  |
|  |  |  |  | All PGD: thyroxine treatment | 0.38 (0.17-0.86) |  |  |
|  |  |  |  | All PGD: thyroxine and methylprednisolone treatment | 0.10 (0.01-0.73) |  |  |
| Nakamura | 2020 | All PGD: Low hemoglobin level (mean: 7.6 g/dL) at donor heart reperfusion | 5.80 (2.32-14.5) |  |  |  |  |
|  |  | All PGD: Middle hemoglobin level (mean: 9.2 g/dL) at donor heart reperfusion | 1.14 (0.40-3.23) |  |  |  |  |
|  |  | All PGD: High hemoglobin level (mean: 10.4 g/dL) at donor heart reperfusion | N/A |  |  |  |  |
| Nicoroa | 2017 | All PGD: african-american race | 1.83 (1.07-3.13) |  |  | All PGD: graft ischemic time (per hour) | 1.80 (1.37-2.42) |
|  |  | All PGD: amiodarone treatment | 1.67 (1.01-2.78) |  |  |  |  |
| Palani | 2021 | All PGD: RADIAL score per unit increase | 5.99 (1.99-18.03) | All PGD: global longitudinal strain on TEE per unit impairment | 3.005 (1.82-4.94) |  |  |
|  |  |  |  | All PGD: ejection fraction | 0.965 (0.796-1.17) |  |  |
| Peled | 2020 | All PGD: statin treatment | 0.35 (0.15-0.81) | All PGD to be preferred due to power-issues. |  |  |  |
|  |  | All PGD: spironolactone treatment | 0.19 (0.09-0.40) |  |  |  |  |
|  |  | All PGD: amiodarone treatment | 3.84 (1.84-8.24) |  |  |  |  |
|  |  | All PGD: ACEi treatment | 0.75 (0.34-1.63) |  |  |  |  |
|  |  | All PGD: male seks | 0.91 (0.22-2.51) |  |  |  |  |
|  |  | All PGD: age (per 5 years) | 0.86 (0.81-1.14) |  |  |  |  |
|  |  | All PGD: ischemic etiology of HF | 1.9 (0.81-4.6) |  |  |  |  |
|  |  | All PGD: Era (HTx after 2000) | 1.38 (0.62-3.09) |  |  |  |  |
|  |  | Moderate-severe PGD: statin treatment | 0.14 (0.02-0.72) |  |  |  |  |
|  |  | Moderate/severe PGD: Spironolactone treatment | 1.71 (0.31-9.73) |  |  |  |  |
|  |  | Moderate/Severe PGD: Amiodarone treatment | 0.62 (0.13-3.01) |  |  |  |  |
|  |  | Moderate/severe PGD: age | 1.08 (1.01-1.16) |  |  |  |  |
|  |  | Moderate/severe PGD: LVAD BTT | 8.09 (2.17-35.24) |  |  |  |  |
| Peled | 2020 | All PGD: female recipient | 0.63 (0.23-1.6) | All PGD: thyroxine treatment | 3.44 (1.26-9.86) |  |  |
|  |  | All PGD: ACEi treatment | 0.73 (0.33-1.61) | All PGD: other hormonal treatment (methylprednisolone and/or vasopressin) | 0.79 (0.34-1.77) |  |  |
|  |  | All PGD: mean pulmonary pressure | 1.02 (0.99-1.05) |  |  |  |  |
| Peled | 2018 | All PGD: pulmonary vascular resistance | 0.91 (0.81-1.22) | All PGD: ethnically non-matched donor | 8.63 (3.12-12.5) | All PGD: ischemic time (per minute) | 1.01 (0.98-1.09) |
| Prieto | 2018 | All PGD: preoperative MCS | 11.90 (2.64-54.12) | sex PGD: female donor | 2.56 (1.29-5.09) | All PGD: total ischemic time (min) | 1.01 (1.00-1.02) |
| Quintana-Quezada | 2016 |  |  | All PGD: Downtime <10 minutes | 0.102 (0.007-1.437) |  |  |
|  |  |  |  | All PGD: Opiate treatment | 4.796 (0.675-34.0) |  |  |
|  |  |  |  | All PGD: male seks | 2.960 (0.549-16.0) |  |  |
|  |  |  |  | All PGD: TBDACC ≥3 days | 0.098 (0.021-0.443) |  |  |
|  |  |  |  | All PGD: TBDACC ≥5 days | 0.092 (0.010-0.411) |  |  |
|  |  |  |  | All PGD: Inotrope score | 0.988 (0.954-1.023) |  |  |
|  |  |  |  | All PGD: mean vasopressin dose | 0.845 (0.229-3.116) |  |  |
|  |  |  |  | All PGD: mean phenylephrine dose | 1.056 (0.369-3.023) |  |  |
|  |  |  |  | All PGD: Death due tonoxia | 4.051 (0.846-19.4) |  |  |
| Ram | 2023 | All PGD: amiodarone pre-HTX | 5.14 (2.52-10.82) | All PGD: combined heart and lungs procurment | 4.63 (2.09-11.04) | All PGD: Ischemic time | 1.01 (1-1.02) |
| Rega | 2024 |  |  |  |  | Severe PGD: static cold storage (compared to HOPE) | 4.2 (1.6-10) |
| Rhee | 2021 | Moderate/severe PGD: preoperative admission | 4.20 (1.24-14.26) |  |  | Moderate/severe PGD: total ischemic time (10-minute increments) | 1.09 (1.02-1.15) |
|  |  | Moderate/severe PGD: preoperative ECMO | 4.03 (1.75-9.26) |  |  |  |  |
| Sabatino | 2017 | Severe PGD: female recipient | 2.84 (1.27-6.16) | Severe PGD: female donor | 2.70 (1.33-5.61) | Severe PGD: cold ischemic time >240 minutes | 3.01 (1.29-6.71) |
|  |  | Severe PGD: congenital heart disease | 11.4 (2.67-45.1) | Severe PGD: Undersized donor | 3.35 (1.23-8.24) |  |  |
|  |  | Severe PGD: pre-transplant anemia | 2.86 (1.06-6.99) |  |  |  |  |
|  |  | Severe PGD: Pre-transplant PVR >3 WU | 2.35 (1.07-5.01) |  |  |  |  |
| Servais | 2024 | Severe PGD: cumulative pre-OHT amiodarone: 3 months | 1.03 (1.001-1.060) | Severe PGD: female donor to male recipient mismatch | 2.558 (0.589-11.111) | Severe PGD: cold ischemic time | 1.006 (0.996-1.016 |
|  |  | Severe PGD: cumulative pre-OHT amiodarone: 6 months | 1.023 (1.003-1.044) | Severe PGD: donor age | 0.978 (0.931-1.028) | Severe PGD: CPB time | 1.012 (1.003-1.022 |
|  |  | Severe PGD: cumulative pre-OHT amiodaron: 12 months | 1.01 (0.998-1.022 | Severe PGD: donor inotrope use at time of OHT | 0.807 (0.245-2.659) | |  |
|  |  | Severe PGD: recipient age | 1.03 (0.977-1.094 |  |  |  |  |
|  |  | severe PGD: LVAD at time of OHT | 0.737 (0.221-2.57) |  |  |  |  |
| Smith | 2021 | All PGD: Age (years) | 1.00 (0.98-1.02) |  |  | All PGD: ischemic time (10 minutes) | 1.05 (1.00-1.10) |
|  |  | All PGD: Prior MCS | 1.66 (0.98-2.80) |  |  | All PGD: perioperative blood products (1 unit) | 1.08 (1.04-1.13) |
|  |  | All PGD: PVR ≥3.5 WU | 1.32 (0.65-2.54) |  |  |  |  |
|  |  | All PGD: Weight mismatch ≥30% | 1.89 (0.65-2.54) |  |  |  |  |
|  |  | All PGD: predicted heart mass mismatch ≥30% | 1.29 (0.29-4.18) |  |  |  |  |
|  |  | All PGD: amiodarone in previous 90 days, 10 gram | 1.10 (0.94-1.27) |  |  |  |  |
|  |  | All PGD: Amiodarone in previous 180 days, 10 gram | 1.05 (0.95-1.15) |  |  |  |  |
| Squiers | 2017 | Moderate/severe PGD: recipient hospitalized | 2.9 (1.0-8.0) | Moderate/severe PGD: undersized donor (≥30% difference in predicted heart mass) | 3.4 (1.1-9.8) |  |  |
|  |  | Moderate/severe PGD: creatinine, per 1.0 mg/dL increase | 5.5 (1.8-19.5) |  |  |  |  |
| Still | 2018 | All PGD: creatinine >2.0 mg/dL | 3.44 (1.23-9.47) |  |  |  |  |
|  |  | All PGD: prior sternotomy | 2.71 (1.36-5.42) |  |  |  |  |
| Takahasi | 2020 |  |  | Moderate/severe PGD: donor age (per year) | 1.10 (1.02-1.17) |  |  |
|  |  |  |  | Moderate/severe PGD: LVEF | 0.82 (0.73-0.92) |  |  |
| Truby | 2021 | Moderate/Severe PGD: Serum C-Type Lectin Domain Family 4 Member C | 1.52 (1.02-3.45) |  |  |  |  |
| Wright | 2017 | Severe PGD: prior VAD | 2.21 (0.84-5.79) | Severe PGD: LVEF | 0.94 (0.88-1.01) | Severe PGD: Ischemic time (hours) | 1.73 (1.10-2.72) |
|  |  | Severe PGD: amiodarone treatment | 6.05 (2.47-14.83) |  |  |  |  |

**Figures**

**Severe PGD**

Figure S1: forest plot of recipient prior sternotomy as a predictor for severe PGD.


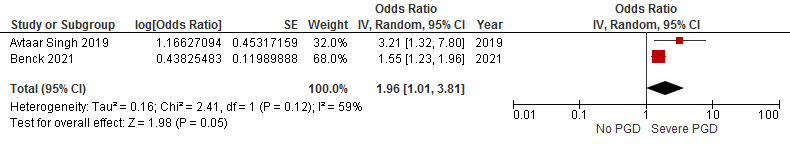


Figure S2: forest plot of recipient left ventricular assist device therapy as a predictor for severe PGD.


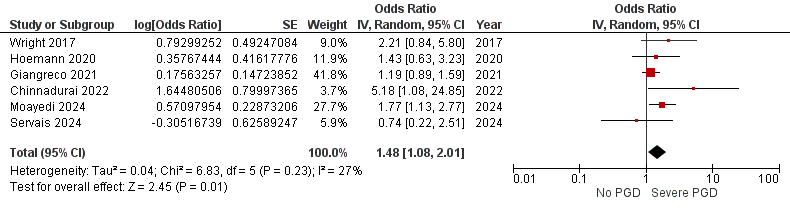


Figure S3: forest plot of recipient amiodarone treatment as a predictor for severe PGD.


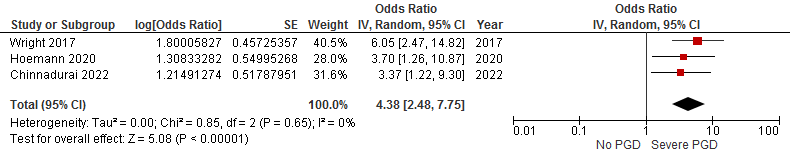


Figure S4: forest plot of female donor as a predictor for severe PGD.


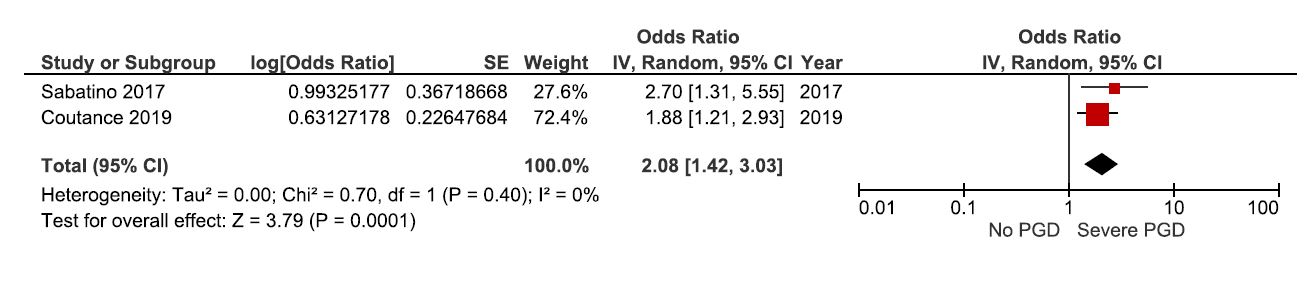


Figure S5: forest plot of cold ischemic time (per hour-increment) as a predictor for severe PGD.


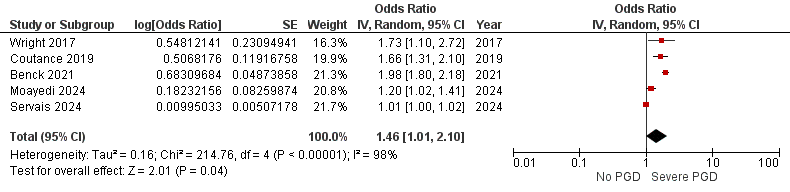


Figure S6: forest plot of blood product (red blood cells, platelets) administration as a predictor for severe PGD.


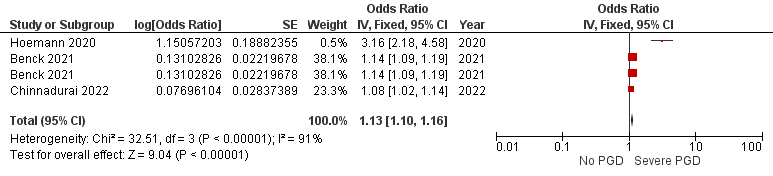

Supplement: Supplementary file 1 — Supplementary material [file mmc1.docx]
